# The impact of poverty-reduction intervention on child mental health mediated by family relations: Findings from a cluster-randomized trial in Uganda

**DOI:** 10.1016/j.socscimed.2023.116102

**Published:** 2023-07-20

**Authors:** Leyla Karimli, Fred M. Ssewamala, Torsten B. Neilands

**Affiliations:** a University of California, Los Angeles (UCLA), Luskin School of Public Affairs, Social Welfare Department, USA; b Washington University in St. Louis, George Warren Brown School of Social Work, USA; c University of California, School of Medicine, San Francisco, USA

**Keywords:** Cluster-randomized controlled trial, Sub-Saharan Africa, AIDS orphans, Child and adolescent mental health, Poverty-reduction interventions, Pathways from poverty to child and adolescent, mental health, Mediation models, Structural equation models (SEM)

## Abstract

Reviews that synthesize global evidence on the impact of poverty reduction interventions on child and adolescent mental health (CAMH) report inconclusive results and highlight the need to unpack the mechanisms that connect poverty-reduction to CAMH. To address this gap, we examine the proposition that family relations is an important relational factor transmitting effect of poverty on CAMH, and test whether family relations mediate the effect of poverty-reduction intervention on depression, hopelessness, and self-concept among AIDS orphans in Uganda.

We use longitudinal data collected over the course of 48 months in a cluster-randomized controlled trial conducted among N = 1410 AIDS orphans from n = 48 schools in Uganda. To examine the relationship between intervention, latent mediator (family relations and support) and CAMH outcomes (Beck Hopelessness Scale (BHS), Tennessee Self-Concept Scale (TSCS), and Depression), we ran structural equation models adjusting for clustering of individuals within schools.

Relative to the control group, participants in both treatment arms reported lower levels of hopelessness and depression, and significantly higher levels of self-concept. They also report significantly higher levels of latent family relationship in all three models. In both treatment arms, the direct effect of the intervention on all three outcomes is still significant when the latent family relations mediator is included in the analyses. This suggests partial mediation. In other words, in both treatment arms, the significant positive effect of the intervention on children’s depression, hopelessness, and self-concept is partially mediated by their family relationship quality.

Our findings support the argument put forward by the Family Stress Model showing that the poverty-reduction program improves children’s mental health functioning by improving family relationships. The implications of our study extend beyond the narrow focus of poverty reduction, suggesting that asset-building interventions have broader impacts on family dynamics and child mental health.

## 1. Introduction

Neuropsychiatric disorders, including depression and anxiety, are among the leading global burden of disease among young people 10–24 years (Mokdad et al., 2016). In Sub-Saharan Africa (SSA), the poorest region in the world (Bicaba et al., 2017; Asongu and Le Roux, 2019) housing the highest number of people living with HIV/AIDS (Dwyer-Lindgren et al., 2019), the combination of poverty and HIV/AIDS increases risks of suboptimal child and adolescent mental health (CAMH) outcomes (Atilola, 2014). The region also has the lowest number of mental health professional per capita, leading to inadequate detention and treatment of mental health disorders among children and adolescents (WHO. Mental health atlas, 2020). A recent systematic review of mental health problems among children and adolescents in SSA (Jörns-Presentati et al., 2021) reports that 26.9% of children and adolescents in the region suffer from depression, 29.8% experience anxiety disorders and symptoms, 40.8% exhibit emotional and behavioral problems and one in 4 children and adolescents express suicidal ideation. Another systematic review focusing on mental health problems of adolescents who are living with HIV in SSA reports that a quarter of this population has some form of a defined psychiatric disorder and 30–50% exhibit emotional and behavioral difficulties (Dessauvagie et al., 2020). In Uganda, studies documenting the prevalence of CAMH disorders are scarce and do not cover national prevalence rates. According to some of these studies, 37% of children and adolescents age 10–14 living with HIV suffer from depressive symptoms (Kemigisha et al., 2019). Moreover, among perinatally HIV-infected children and adolescents age 5–17 in Uganda, 17% of them report having at least one psychiatric disorder and 4% report having a major depressive disorder (Kinyanda et al., 2019).

Looking into social determinants of health and mental health, multiple studies have identified poverty as a primary risk factor for impaired mental health functioning in children and adolescents (Lund et al., 2018; Yoshikawa et al., 2012). Research has documented that children and adolescents who live in poor households, and therefore experience financial strain, are more likely to suffer from poor self-esteem, low self-efficacy, high depression, increased anxiety, and reduced life satisfaction (Kim et al., 2019; Ridley et al., 2020). Within this context, due to the differential exposure to stressors based on gender, adolescent girls may be at a higher risk of experiencing adverse mental health outcomes compared with their male peers (Campbell et al., 2021). Furthermore, children and adolescents who have lost a parent to AIDS—hence referred to as AIDS orphaned children—are at a greater risk of poverty, which, in combination with family disruption and reduced social and family support, pose a greater risk for these children’s mental health (Jörns-Presentati et al., 2021; Lata and Verma, 2013; Ashaba et al., 2019). Studies found that AIDS-orphaned children and adolescents are more likely to report higher levels of anxiety, depression, sadness, hopelessness, and peer relationship problems, compared to non-orphans (Cluver et al., 2007; Atwine et al., 2005).

Identifying poverty as a significant risk factor for child and adolescent mental health has prompted development of poverty-reduction interventions that seek to improve child wellbeing by mitigating the effects of poverty. Despite these efforts, however, evidence about the effect of poverty-reduction interventions on CAMH is inconclusive (Zaneva et al., 2022; Zimmerman et al., 2021). A review of cash programs in low and middle-income countries (LMICs) has found that these programs had a positive effect on some externalizing symptoms in children and adolescents, while having no effect on depressive symptoms (Zimmerman et al., 2021). However, the small number of studies and significant heterogeneity across them are cited as potential factors that may have contributed to this finding. Another review of cash and grant programs that includes not only LMICs, but also high-income countries and covers a broader range of internalizing symptoms (Zaneva et al., 2022), reports overall positive effect of poverty alleviation programs on internalizing symptoms among children and adolescents. Both reviews suggest that the effect of poverty-reduction interventions on CAMH is contingent on whether these interventions address the underlying socioeconomic conditions, including family relationships. This highlights the crucial need for a more comprehensive exploration of the intricate pathways and underlying mechanisms that connect poverty-reduction programs to CAMH.

Examining the relationship between poverty and mental health, a social causation theory (Link and Phelan, 1995) suggests that adverse living conditions associated with poverty and poverty-related stressors, such as poor housing conditions, financial insecurity, and exposure to violence, can lead to significant mental health problems in both children and adults. However, children who live in poverty are exposed to suboptimal levels of not only health, nutrition, and learning, but also relationships, security, and safety (Victora et al., 2022). It is important to note that the pathways through which poverty affects CAMH include not only biological (e.g., nutrition deficiencies, infectious diseases) and sociocultural determinants (e.g., reduced access to health services, low maternal education), but also psychosocial factors, such as parenting and family relationships (Walker et al., 2007).

A theoretical framework of the Family Stress Model (Conger et al., 2010) posits that the link from poverty to child mental health is shaped by family relationships, which can play a crucial role in the transmission of stress as well as the promotion of resilience. Within this framework, the quality of family relationships is considered one of the most important relational factors that can help mitigate the effect of poverty on CAMH (Yoshikawa et al., 2012). According to the Family Stress Model, socioeconomic deprivation and economic hardships lead to parents’ psychological distress and, consequently, to deteriorated family cohesion, disruptive parenting, and reduced quality and quantity of time spent with children, which, in turn, results in child and adolescent maladjustment, including internalizing and externalizing disorders (Masarik and Conger, 2017; Gard et al., 2020). Conversely, ongoing positive relationships with a caregiver play a crucial role in fostering a child’s resilience; and resources like family support and adult mentoring can help children and adolescents overcome the negative effects of risk exposure, effectively manage traumatic experiences, and avoid negative trajectories commonly associated with risk factors (Fergus and Zimmerman, 2005; Yule et al., 2019).

We place our study within the theoretical framework of the Family Stress Model and examine a type of poverty-reduction intervention—called Bridges to the Future (abbreviated as Bridges)—where children are the explicit beneficiaries of the program. This intervention supports modest accumulation of financial assets in matched child development accounts (also known as CDAs) and integrates the financial component with the family strengthening component. CDAs have the potential to significantly mitigate the unique challenges faced by AIDS orphans who have lost their parents. These children face not only the emotional and psychological impact of losing their parents but also are at a greater risk of poverty and socioeconomic instability (Jörns-Presentati et al., 2021; Lata and Verma, 2013; Ashaba et al., 2019). CDA can serve as a valuable tool in addressing the economic vulnerabilities and uncertainties faced by these children, potentially reducing their exposure to poverty, and enhancing their overall well-being. Indeed, multiple studies have shown that CDAs have significant positive impact on the psychological wellbeing and mental health functioning of AIDS-orphaned children and adolescents (Ssewamala et al., 2016, 2021, 2023).

Prior research has examined different pathways through which these interventions may affect CAMH. For example, previous studies have explored whether accumulated savings (Karimli and Ssewamala, 2015), reduced child poverty and labor, and improved household wealth (Karimli et al., 2019) might serve as mechanisms that mediate the relationship between the intervention and CAMH outcomes. Despite these efforts, to the best of our knowledge, no studies have yet examined the role of family relationships as a potential pathway to explain the effect of child savings accounts on CAMH.

Situated within the theoretical framework of the Family Stress Model, our study hypothesizes that the quality of the family relationship will mediate the observable effect of the asset-based poverty-reduction intervention on children’s mental health functioning. By exploring the role of family relationship as a mechanism through which poverty-reduction interventions affect child mental health, we aim to contribute to a deeper and more nuanced understanding of the complex and dynamic interplay between poverty, family relationships and CAMH.

## 2. Methods

### 2.1. Study design

We utilized data from a 5-year (2011–2016) National Institutes of Health (NIH)-funded longitudinal randomized controlled trial (please see CONSORT chart attached). A total of 1410- children (n = 621 boys and n = 789 girls), with mean age of 12.7 years at enrollment (range 10–16 years) were recruited to participate in the study. Children were eligible to participate if they: 1) had lost one or both parents to AIDS, 2) were living within a family, not in an institution, and 3) were enrolled in a government-aided public school in primary 5 or 6 (an equivalent of 6th or 7th grades in the U.S. educational system). Participants were recruited from 48 rural primary schools, in 4 districts of Rakai, Masaka, Lwengo and Kalungu in south-central Uganda, a region heavily affected by HIV/AIDS, with a HIV prevalence range of 11.7% compared with national average of 5.4% (Uganda Aids Commission Secretariat, 2021).

Each of the 48 primary schools was randomly assigned to either the control arm (n = 16 schools, 496 participants) or one of the 2 two treatment arms: Bridges arm (n = 16 schools, 402 participants) or Bridges PLUS arm (n = 16 schools, 512 participants). Randomization was conducted at the school level to minimize cross-arm contamination. Participants in the control arm received usual care services offered to orphaned children and adolescents in the region. Usual care includes counseling (typically conducted by church pastors), food aid (in the form of school lunches), and scholastic materials (such as textbooks, notebooks, and school uniforms). Participants in the two treatment arms: Bridges and Bridges PLUS, received the usual care mentioned above plus 3 intervention components: 1) a CDA – child development account, a family economic strengthening intervention. The CDA was held in both the child and caregiver’s name in a recognized financial institution. The accumulated savings in a CDA were matched with money from the program by a ratio of 1:1 for the Bridges arm and a 2:1 match ratio for the Bridges PLUS arm. The match rate was the only difference between the two treatment arms. The matched CDA was intended to pay for the child’s post primary education and/or start a microenterprise business; 2) Financial Literacy Training, including sessions on saving and microenterprise development was provided to both treatment arms; and 3) In addition, all participants in the treatment arms received mentorship guided by a 9-session curriculum. The mentorship program was intended to help participants to develop the ability to identify specific future goals and educational aspirations.

Longitudinal data were collected from children and adolescents at five data points: baseline, 12 months, 24 months, 36 months, and 48 months post intervention initiation. Data were collected using a 90-min structured survey, administered by trained Ugandan interviewers. Each interviewer had to undergo good clinical practice training and had to obtain a Collaborative Institutional Training Initiative (CITI) research ethics training certificate before interacting with study participants.

### 2.2. Measures

All measures have been tested in previous studies among children and adolescents affected by AIDS in Uganda (Ssewamala et al., 2016, 2021).

#### Outcomes.

Child and adolescent mental health were captured using three measures: Child Depression Inventory, The Beck Hopelessness Scale, and Tennessee Self-Concept Scale.

1. *The Child Depression Inventory* is a 27-item scale assessing children’s depressive symptoms. Each item has three response options that correspond to varying levels of symptomology for clinical depression. The scale ranges from 0 to 81, with high scores indicting high levels of adolescents’ depressive symptoms. The scale has moderate internal reliability (Cronbach’s alpha ranged from 0.66 at baseline to 0.65 at Wave 5).
2. *The Beck Hopelessness Scale* is a 20-item scale that measures hopelessness and pessimistic attitudes toward the future. Items are binary coded. The scale ranges from 0 to 20, with high scores indicting high levels of adolescents’ hopelessness. The scale has moderate to high internal reliability (Cronbach’s alpha ranged from is 0.65 at baseline to 0.76 at wave 5).
3. *The Tennessee Self-Concept Scale* is a 20-item scale that measures children’s perception of identity, self-satisfaction, and other behaviors on a 5-point scale (1 always false to 5 always true). The scale ranges from 0 to 80, with high scores indicting high levels of ado- lescents’ self-concept. The scale has high internal reliability (Cronbach’s alpha ranged from is 0.72 at baseline to 0.82 at wave 5)

#### Mediator.

To reduce the effects of measurement error, account for correlations among multiple observed scales measuring the same latent construct, and to maximize statistical power for tests of mediation (Hoyle et al., 1999) we constructed a latent variable for the mediator: family relationships. Consistent with previous research using the Family Assessment Measure (FAM) and the Family Environment Scale (FES) to evaluate family relationships (Booysen et al., 2021), we constructed a latent variable of family relations using the three indicators described below.

1. *Family cohesion,* using the Family Environment Scale (Moos and Moos, 1994) and the Family Assessment Measure (Skinner et al., 2009), was assessed using eight items that measure the degree of help, commitment and support between the family members. Participants were asked to rate how often each item occurs in their family, on a 5-point scale (0 Never to 4 Always). The scale ranges from 0 to 32, with high scores indicating high levels of family cohesion. The scale has high internal reliability (Cronbach’s alpha = 0.76)
2. *Child-caregiver relationship* was assessed by asking children 18 questions to rate relationships with the adults they live with, on a 5-likert scale (0 = Never and 4 = Always). Examples of questions adapted from the Social Support Behavior Scale (SS–B) (Vaux et al., 1987) include “*Do your current parent/guardian show interest in your schoolwork*”, “*Do your current parent/guardian act cold or unfriendly if you do something they don’t like*”, “*Do your parent/guardian spend time just talking with you*?“. The score ranges from 0 to 72. High scores represent high quality of child-caregiver relationship. The scale has high internal reliability (Cronbach’s alpha 0.75).
3. *Perceived caregiver support* was measured using six items adapted from the Family Environment Scale (Moos and Moos, 1994) and the Family Assessment Measure (Skinner et al., 2009) asking children to react to statements about their parents understating them, wanting to hear about their problems, caring about their feelings, treating them like they really matter, liking them the way they are, and acting like they do is important. Each of these 6 items was measured on a 5-likert scale (0 = Never and 4 = Always).

#### Intervention.

As described earlier, intervention had two treatment arms with the match rate (i.e., financial incentive for saving) being the only difference between the arms: in Bridges arm, the accumulated savings in a CDA were matched by a ratio of 1:1, while in Bridges PLUS arm the math ratio was 2:1. Our analyses examine direct and mediating effects for each of the two treatment arms.

#### Covariates.

All models control for child’s orphanhood status (single paternal orphan, single maternal orphans, double orphan), child’s gender (boys, girls), and child’s age.

### 2.3. Statistical analyses procedures

We follow the CONSORT guideline to report the baseline sample characteristics. To examine the individual-level variations in outcomes while accounting for potential between-school correlations, we report adjusted Wald F-statistics (design-based F).

**Fig. 1.**
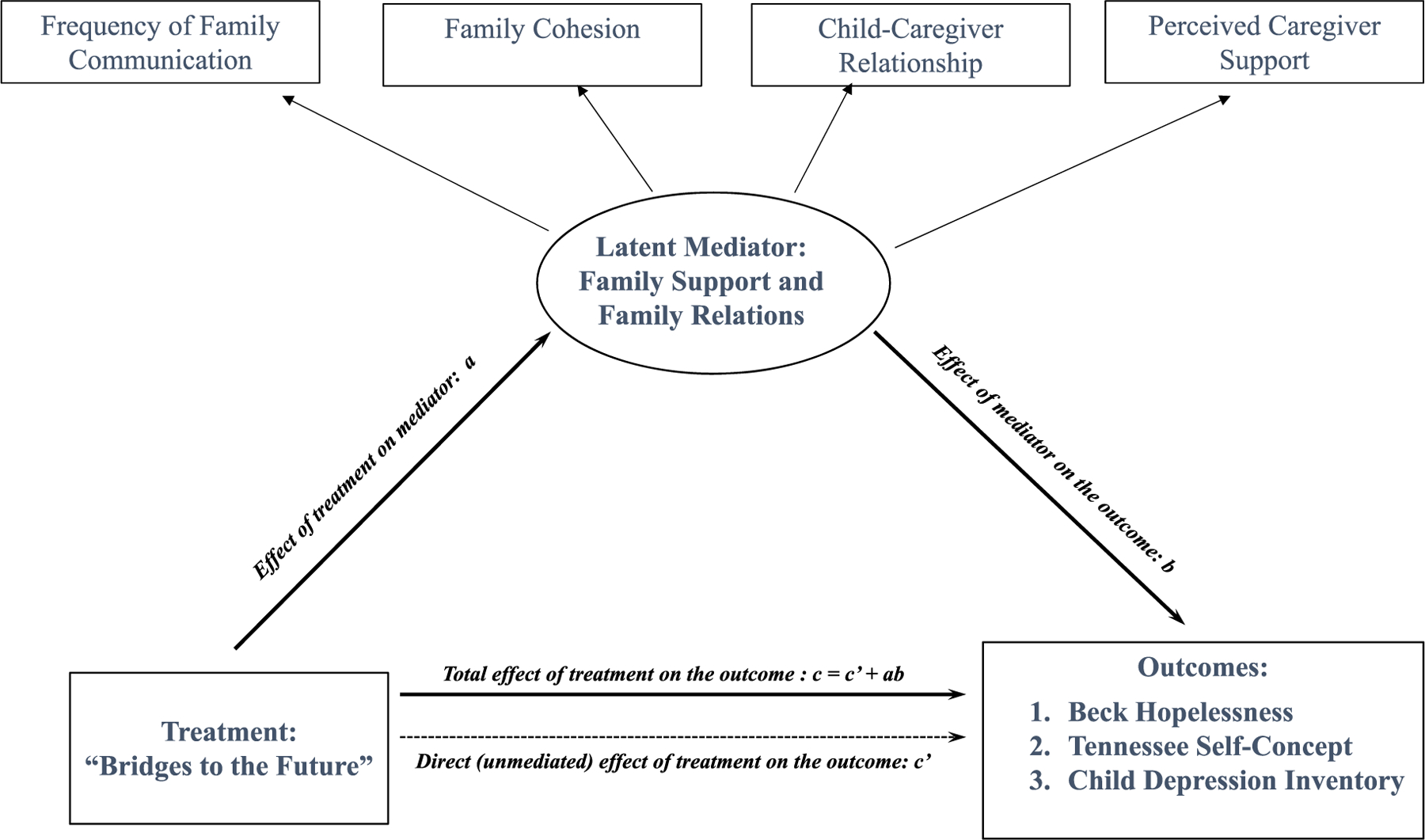
Structural Equation Model.

To answer our research question, for each of the three outcome variables, we fitted three mediation models using M*plus* 8.9. In these models (Fig. 1), (i) the direct effect (*c’*) is the pathway from the intervention to the outcome while controlling for the mediator (i.e., unmediated effect of intervention); (ii) the indirect effect (a*b) is the pathway from the intervention to the outcome through mediator; and (iii) the total effect (*c*) is the sum of direct and indirect effects (*c = c’ + ab*) (Hair et al., 2021).

To avoid potential bias that may occur from not including all the time points’ mediators and outcomes in cross-sectional or sequential mediation, we use cross-lagged autoregressive mediation models that incorporate mediators and outcomes at all time points (Mitchell and Maxwell, 2013). In these models, each of the three outcomes is regressed at each time point onto the intervention (dummy variables created for each group) and the preceding time point’s outcome. The latent mediator is regressed onto the intervention and the preceding time point’s mediator. Residuals for the latent mediator and outcomes within the same time point are allowed to correlate, while the regression coefficients, residual variances, and correlations among residuals are set equal across time (Mitchell and Maxwell, 2013). The factor loadings and intercepts of the latent mediator’s indicators were set equal across time points to establish scalar invariance of the latent mediator over time (Millsap et al., 2012).

To evaluate the global model fit of our models, we report the chi-square test of model fit, the Root Mean Square Error of Approximation (RMSEA), and the Standardized Root Mean Square Residual (SRMR) (Kline, 2015), using RMSEA 0.06 and SRMR <0.08 as a threshold for satisfactory model fit (Hu and Bentler, 1999).

Standard errors and test statistics adjusted for clustering of individuals within schools using robust Huber-White sandwich variance estimation. Additionally, in order to account for asymmetric distributions of the indirect effects, we report cluster-adjusted bootstrap-based confidence intervals with 5000 requested replicate samples for direct, indirect, and total effects (MacKinnon et al., 2004). The effect is considered to be significant at *p* < 0.05 if the bootstrap-based 95% CI excludes zero.

## 3. Results

Table 1 describes baseline characteristics of our sample.

At baseline, about 19.6% of children and adolescents in the study were double orphans (i.e., children who have lost both biological parents). On average, participants were 13 years old, and about 56% of them were female children and adolescents. The average depression score among the study participants was 9.8 out of 48, the average score on the Beck Hopelessness Scale was 5.4 out of 20, and the average score on Tennessee Self-Concept Scale was 49.8 out of 80.

Participants in the Bridges arm (with savings matched on a ratio of 1:1) reported significantly lower levels of hopelessness (B = −0.406; 95% CI = −0.644, −0.167) and depression (B = −0.412; 95% CI = −0.726, −0.098), and significantly higher levels of self-concept (B = 0.795, 95% CI = 0.286, 1.304) compared with their control group counterparts. Similarly, participants in the Bridges PLUS treatment arm (with savings matched on a ratio of 2:1) reported significantly lower levels of hopelessness (B = −0.478; 95% CI = −0.681, −0.275) and depression (B = −0.444; 95% CI = −0.755, −0.133), and significantly higher levels of self-concept (B = 0.788; 95% CI = 0.255,1.321) compared with their control group counterparts.

Relative to the control group, participants in both treatment arms reported higher levels of latent family relationship in all three models (Table 2). In other words, the mediator’s effect on the outcome (component *b* in the *c = c’ + ab* formula mentioned above) is statistically significant for each of the three outcomes. Furthermore, the indirect effects (i.e., the pathway from the intervention to the outcome through the mediator, component *ab* in the *c = c’ + ab* formula described above) are statistically significant for all three outcomes. In particular, the indirect effects show that improved family relationships resulted in lower levels of subsequent depression and hopelessness and higher levels of subsequent self-concept for participants in both treatment arms (Table 2).

In both treatment arms, the direct effect of the intervention on all three outcomes is still significant when the latent family relations mediator is included in the analyses. This suggests partial mediation. In other words, in both treatment arms, the significant positive effect of the Bridges intervention on children’s depression, hopelessness, and self-concept is partially mediated by their family relationship quality.

## 4. Discussion

The current study contributes to understanding the mechanisms through which poverty-reduction interventions impact child and adolescent mental health. Poverty can have detrimental effects on child development, including cognitive, emotional, and behavioral difficulties (Lund et al., 2018; Yoshikawa et al., 2012; Ridley et al., 2020). These challenges can have lifelong consequences and contribute to a cycle of poverty that persists across generations (Bird et al., 2013; Attanasio et al., 2022). Effective poverty-reduction interventions that improve child mental health can help break this cycle. However, the impact of poverty-reduction interventions on CAMH can vary widely, depending on a range of factors, including family relationships (Zaneva et al., 2022; Zimmerman et al., 2021). Family relationships can play a crucial role in shaping children’s development (Fergus and Zimmerman, 2005; Yule et al., 2019). Therefore, understanding the role of family relationships in transmitting the effect of poverty-reduction interventions on CAMH can help the field to develop more tailored interventions that address the underlying social and environmental conditions related to poor mental health outcomes in children.

Our study is grounded in the Family Stress Model, which proposes that poverty adversely affects child mental health by undermining family functioning and family relationships, and, consequently, poverty-reduction interventions may improve CAMH by improving family functioning and family relationships. Despite the increasing recognition of interconnections among socioeconomic status, family functioning, and CAMH (Ridley et al., 2020), very few studies have offered robust empirical evidence of family relationships as pathways through which poverty-reduction programs affect CAMH. Given the well-documented dearth of information on this topic (Zaneva et al., 2022; Zimmerman et al., 2021), the current study represents a significant contribution to the body of knowledge to expand our understanding of underlying socioeconomic conditions and unpacking the pathways that link poverty to CAMH.

We found strong evidence that family relationships, as reported by children and adolescents, partially mediated the effect of the Bridges asset-based poverty-reduction intervention on reducing children and adolescents’ depression and hopelessness and improving children and adolescents’ self-concepts. In other words, we found that improved family relationships among the treatment group participants resulted in lower levels of subsequent depression and hopelessness and higher levels of subsequent self-concept.

Our findings support the argument put forward by the Family Stress Model (Conger et al., 2010; Masarik and Conger, 2017) showing that the poverty-reduction program improves children’s mental health functioning by improving family relationships. It reinforces the importance of relational factors, such as quality of family relationships, as pathways between poverty and CAMH (Yoshikawa et al., 2012). It also suggests that the quality of family relationships and family support, as a specific type of social support, can be a vital adaptive resource and protective factor strengthening child and adolescent resilience and, thus, reducing effect of poverty on CAMH (Masten, 2018). In particular, for children and adolescents orphaned by AIDS, family support (offered by a surviving parent, aunts, uncles, or grandparents) may help address mental health functioning by providing the psychological and material support resources needed to cope with stress related to orphanhood and poverty (Yule et al., 2019; Haroz et al., 2013; Okawa et al., 2011).

One of the key limitations of this study is its reliance on self-reported measures of child mental health. Although the psychometric rigor and utility of these measures have been widely accepted, we acknowledge a call for caution in claiming a causal nature of reported associations. Furthermore, we refrain from using cutoff points and the diagnostic criteria prescribed in the DSM (Diagnostic and Statistical Manual of Mental Disorders) because they often inadequately capture cross-cultural variation in mental health experiences (Haroz et al., 2017) and due to potential cross-cultural differences in somatization of mental health issues (Ali et al., 2016). We treated children’s mental health as a continuum such that even subclinical deficits in mental health may lower quality of life and may be a public health concern (Karimli et al., 2019). Finally, while our longitudinal mediation analysis approach eliminated the bias arising in the analysis of longitudinal data from the application of classic three-variable sequential mediation models, and thus represents a step forward, unmeasured residual confounding between the mediator and outcomes remains a possibility as is the case in any study in which the mediator cannot be randomized.

Our study provides valuable insights into the mechanisms by which poverty-reduction interventions based on asset accumulation can enhance the mental health of children and adolescents in low-income settings. Our study brings a unique perspective to the field, suggesting that interventions focused on asset accumulation—designed to build resources for a child’s future and involving the entire family—can be considered a multifaceted intervention that has broader impacts on family well-being, rather than solely a poverty-reduction strategy. Our findings highlight the potential for such interventions to not only reduce poverty, but also enhance family functioning and family relationships, leading to positive mental health outcomes for children and adolescents. The implications of our study extend beyond the narrow focus of poverty reduction, suggesting that asset-building interventions have broader impacts on family dynamics and child mental health.

## Figures and Tables

**Figure:**
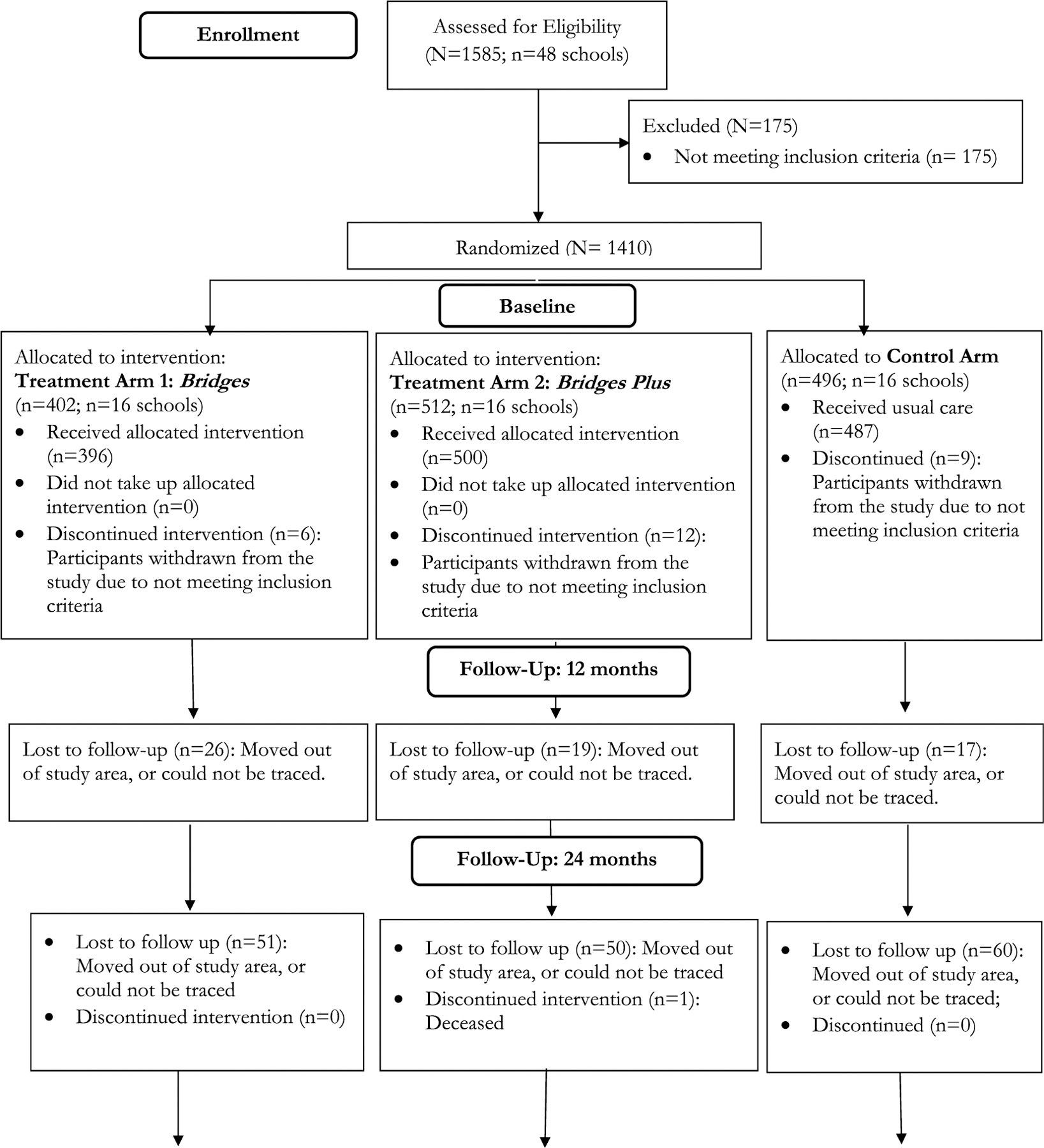
The CONSORT study flow chart

**Table 1:** Baseline characteristics of sample.

VARIABLES	Control Group (n = 496)		Treatment Arm 1 (n = 402)		Treatment Arm 2 (n = 512)		Total (n = 1410)		Design-based F
	
	Percentage or Mean [95% Confidence Intervals)								
OUTCOMES									
Depression (Range: 0–48)	9.7	[9.1; 10.4]	9.7	[9.1; 10.3]	9.99	[9.3; 10.7]	9.8	[9.4; 10.2]	0.22
Tennessee Self-Concept Scale (0–80)	50.4	[49.6; 51]	50.5	[49; 51.7]	48.7	[47.5; 49.8]	49.8	[49; 50.5]	3.61*
Beck Hopelessness Scale (0–20)	5.4	[5, 5.7]	5	[4.9, 5.4]	5.5	[5.1, 5.9]	5.4	[5.2, 5.6]	1.54
MEDIATORS									
Family cohesion (Range: 0–32)	24	[23; 25]	23	[22.5; 24]	23	[22.6; 24]	23.6	[23; 24]	0.43
Child-caregiver relationship (Range: 0–72)	42.6	[42; 43]	44	[43.4; 44.6]	42.9	[42; 43.6]	43.0	[42.7; 43.5]	6.09**
Perceived caregiver support (Range: 0–24)	18	[17.8; 18.6]	17.6	[17; 18]	17.8	[17; 18]	17.9	[17.6; 18]	1.5
DEMOGRAPHICS									
Child’s age (Range: 9–17)	12.75	[12.6; 12.9]	12.56	[12.4; 12.7]	12.71	[12.6; 12.8]	12.68	[12.6; 12.8]	1.33
Child’s orphanhood status (%)									1.79
*Double orphan*	22.8	[19.9; 26]	15.9	[12.2; 20.5]	19.3	[15.2; 24.3]	19.6	[17.2; 22.1]	
*Single paternal orphan*	22.8	[17.3; 29.3]	20.2	[16.5; 24.4]	22.3	[18.3; 26.8]	21.8	[19.1; 24.9]	
*Single maternal orphan*	54.4	[49.2; 59.6]	63.9	[58; 69.5]	58.4	[55.2; 61.5]	58.6	[55.6; 61.5]	
Child’s gender (%)									0.12
*Male*	44.96	[40.4; 49.6]	43.28	[36.9; 49.9]	43.75	[39.8; 47.8]	44.04	[41.2; 46.9]	
*Female*	55.04	[50.4; 59.6]	56.72	[50.1; 63.1]	56.25	[52.2; 60.2]	55.96	[53.1; 58.8]	
Household structure									
*Number of months child lived at their current home (Range: 0*–*192)*	85.93	[81.2; 90.7]	85.79	[80.2; 91.4]	89.49	[83.3; 95.7]	87.18	[83.8; 90.5]	0.5
*Number of people in the household (Range: 0*–*21)*	6	[6.3; 6.7]	6	[6; 6.6]	6	[6.1; 6.6]	6	[6.2; 6.5]	0.76
*Number of children in the household (Range: 0*–*19)*	3	[3; 3.4]	3	[2.9; 3.3]	3	[3; 3.5]	3	[3; 3.3]	0.29
How many sets of clothes child has?									0.88
*None*	0.4	[0.1,1.4]	0.2	[0.0,1.6]	0		0.2	[0.1,0.6]	
*One*	3.8	[2.5,5.9]	4.2	[2.2,8.0]	5.3	[3.5,8.0]	4.5	[3.3,5.9]	
*Two*	11.1	[7.3,16.4]	9.7	[6.9,13.5]	7.4	[4.9,11.1]	9.4	[7.4,11.8]	
*More than two*	84.7	[79.4,88.8]	85.8	[80.5,89.9]	87.3	[82.9,90.7]	86	[83.2,88.4]	
Child has a blanket									1.59
*No*	34.9	[25.9,45.1]	26.1	[19.5,34.0]	34.4	[29.8,39.3]	32.2	[27.8,36.9]	
*Yes*	65.1	[54.9,74.1]	73.9	[66.0,80.5]	65.6	[60.7,70.2]	67.8	[63.1,72.2]	
How many pairs of shoes child has?									2.55*
*None*	36.5	[26.5,47.8]	23.1	[17.2,30.3]	28.3	[24.5,32.5]	29.7	[25.2,34.7]	
*One*	45.4	[38.6,52.3]	55.2	[49.6,60.7]	56.3	[52.4,60.1]	52.1	[48.6,55.7]	
*Two*	14.7	[10.0,21.1]	16.4	[12.3,21.6]	10	[7.7,12.8]	13.5	[11.0,16.4]	
*More than two*	3.4	[1.8,6.4]	5.2	[2.8,9.5]	5.5	[3.3,9.0]	4.7	[3.3,6.5]	
Average number of meals child took per day in the last 7 days									0.54
*None*	0.2	[0.0,1.4]	0.5	[0.1,1.7]	0.2	[0.0,1.4]	0.3	[0.1,0.7]	
*One*	16.5	[12.1,22.1]	21.1	[15.3,28.5]	18.6	[14.0,24.2]	18.6	[15.5,22.1]	
*Two*	64.5	[59.9,68.9]	58.5	[53.6,63.2]	62.5	[57.5,67.3]	62.1	[59.1,64.9]	
*Three*	18.8	[14.7,23.7]	19.9	[15.5,25.2]	18.8	[14.1,24.5]	19.1	[16.4,22.0]	
Child currently works for pay									0.05
*No*	89.9	[86.0,92.8]	90.3	[85.4,93.7]	90.6	[87.1,93.3]	90.3	[88.1,92.1]	
*Yes*	10.1	[7.2,14.0]	9.7	[6.3,14.6]	9.4	[6.7,12.9]	9.7	[7.9,11.9]	
Does the house have electricity?									2.00
*No*	93.8	[89.5,96.4]	87.1	[79.4,92.2]	90.6	[84.9,94.3]	90.7	[87.4,93.2]	
*Yes*	6.3	[3.6,10.5]	12.9	[7.8,20.6]	9.4	[5.7,15.1]	9.3	[6.8,12.6]	
What kind of house do you live in?									1.44
*Muzigo*	4.2	[2.7,6.7]	7.5	[4.5,12.1]	6.6	[4.0,10.9]	6	[4.4,8.1]	
*Hut*	0.2	[0.0,1.5]	0.5	[0.1,2.0]	1.6	[0.7,3.4]	0.8	[0.4,1.5]	
*Mud house*	24	[19.1,29.7]	16.9	[11.8,23.6]	22.1	[14.4,32.3]	21.3	[17.4,25.8]	
*Brick house with iron sheets but not cemented floors*	35.4	[29.3,41.9]	30.3	[24.8,36.5]	30.3	[26.1,34.8]	32.1	[28.9,35.4]	
*Brick house with iron sheets and cemented floors*	36.2	[29.9,42.9]	44.8	[37.4,52.3]	39.5	[31.8,47.7]	39.8	[35.5,44.3]	
How far is it from your home to your primary school?									0.65
*Very near (about 0-*1 km*, you would walk)*	64.5	[56.8,71.5]	59.9	[51.5,67.8]	66.1	[59.9,71.8]	63.8	[59.4,67.9]	
*Near (about 1-*3 kms*, maybe you would use a bicycle)*	34.4	[27.9,41.5]	39.3	[31.7,47.3]	32.6	[27.9,37.8]	35.2	[31.3,39.2]	
*Far (over* 3 kms*, you would use a boda boda (a motorcycle taxi))*	0.6	[0.1,3.0]	0		0.6	[0.2,1.9]	0.5	[0.2,1.2]	
*Very far (you would need a car)*	0.4	[0.1,1.6]	0.8	[0.2,3.2]	0.6	[0.1,4.5]	0.6	[0.2,1.6]	
How far is the public secondary school from your home?									1.38
*Very near (about 0-*1 km*, you would walk)*	17.7	[11.6,26.2]	28	[21.4,35.7]	19.5	[12.8,28.6]	21.4	[17.2,26.2]	
*Near (about 1-*3 kms*, maybe you would use a bicycle)*	21.4	[14.9,29.8]	23.4	[17.9,29.8]	22.6	[18.0,28.0]	22.4	[19.0,26.2]	
*Far (over* 3 kms*, you would use a boda boda (a motorcycle taxi))*	27.2	[20.2,35.5]	28	[21.4,35.7]	26.4	[22.0,31.3]	27.1	[23.5,31.1]	
*Very far (you would need a car)*	33.6	[24.6,44.0]	20.6	[14.5,28.4]	31.5	[23.2,41.2]	29.1	[24.0,34.7]	
How far is the health clinic from your home?									0.70
*Very near (about 0-*1 km*, you would walk)*	36.6	[30.4,43.3]	43.1	[36.0,50.4]	39	[33.5,44.9]	39.4	[35.6,43.2]	
*Near (about 1-*3 kms*, maybe you would use a bicycle)*	15.9	[11.8,21.1]	17.2	[13.8,21.4]	14.6	[10.9,19.3]	15.8	[13.5,18.4]	
*Far (over* 3 kms*, you would use a boda boda (a motorcycle taxi))*	9.2	[6.7,12.4]	8.1	[5.8,11.1]	10	[8.3,12.1]	9.2	[7.8,10.7]	
*Very far (you would need a car)*	38.3	[30.4,46.9]	31.6	[24.6,39.5]	36.3	[29.8,43.4]	35.7	[31.3,40.3]	
How far is the hospital from your home?									0.35
*Very near (about 0-*1 km*, you would walk)*	5.4	[1.7,15.9]	4.3	[1.6,11.1]	3	[1.1,7.9]	4.2	[2.2,8.0]	
*Near (about 1-*3 kms*, maybe you would use a bicycle)*	6.2	[2.5,14.7]	7.3	[3.6,14.2]	5.9	[2.9,11.3]	6.4	[4.1,9.8]	
*Far (over* 3 kms*, you would use a boda boda (a motorcycle taxi))*	11.6	[6.1,21.1]	17.1	[10.8,25.9]	12.4	[7.8,19.2]	13.5	[9.9,18.0]	
*Very far (you would need a car)*	76.7	[55.8,89.6]	71.3	[56.0,83.0]	78.7	[66.2,87.4]	75.9	[66.8,83.2]	
How far is the nearest water source (well, lap water, borehole) from your home?									1.61
*Very near (about 0-*1 km*, you would walk)*	68.6	[62.1,74.5]	72.4	[68.4,76.1]	65.1	[59.4,70.5]	68.4	[65.0,71.6]	
*Near (about 1-*3 kms*, maybe you would use a bicycle)*	18.7	[14.4,23.9]	16.2	[13.2,19.7]	21.2	[16.8,26.3]	18.9	[16.4,21.6]	
*Far (over* 3 kms*, you would use a boda boda (a motorcycle taxi))*	10.8	[7.7,14.8]	10.6	[7.3,15.1]	10.2	[7.9,13.0]	10.5	[8.8,12.5]	
*Very far (you would need a car)*	1.9	[0.9,4.1]	0.8	[0.3,2.1]	3.5	[1.9,6.4]	2.2	[1.4,3.5]	

**Boldface** type indicates statistically significant results.

* p ≤ 0.05,

** p ≤ 0.01

*** p ≤ 0.001.

We report adjusted Wald F-statistics **(Design-based F)** to examine individual-level variations while accounting for potential correlation between same-school observations.

**Table 2:** Structural equation model (SEM) results: 24 Months follow-up.

	Beck Hopelessness Scale		Child Depression Inventory		Tennessee Self-Concept Scale	
**Global Model Fit**						
Chi-square test of model fit (χ^2^ (DF), p)	1279.8 (287), <0.000		1411.9 (287), <0.000		1198.9 (287), <0.000	
SRMR (Standardized Root Mean Square Residual)	0.06		0.08		0.06	
RMSEA (Root Mean Square Error Of Approximation) (Estimate [95% CI])	0.05	[0.047; 0.052]	0.05	[0.05; 0.055]	0.05	[0.045; 0.05]

**Direct Effects**	**B**	**[95% CI]**	**B**	**[95% CI]**	**B**	**[95% CI]**

**Mediator (Time T)**						
Treatment Arm 1 (Time T-1)	0.243*	[0.049; 0.436]	0.216*	[0.023; 0.409]	0.241*	[0.039; 0.443]
Treatment Arm 2 (Time T-1)	0.257*	[0.070; 0.445]	0.234*	[0.045; 0.423]	0.243*	[0.045; 0.441]
Mediator (Time T-1)	0.858*	[0.813; 0.902]	0.857*	[0.813; 0.901]	0.836*	[0.797; 0.875]
Outcome (Time T-1)	0.061*	[0.017; 0.105]	0.038*	[0.011; 0.064]	−0.013*	[−0.023; −0.002]
Covariates						
*Child’s gender*	0.007	[−0.145; 0.160]	0.007	[−0.152; 0.165]	0.153	[−0.002; 0.309]
*Child’s age*	−0.044*	[−0.068; −0.019]	−0.05*	[−0.073; −0.026]	0.007	[−0.034; 0.047]
*Child’s orphanhood status*	0.209*	[0.126; 0.291]	0.211*	[0.127; 0.295]	0.242*	[0.155; 0.328]
**Outcome (Time T)**						
Treatment Arm 1 (Time T-1)	−0.406*	[−0.644; −0.167]	−0.412*	[−0.726; −0.098]	0.795*	[0.286; 1.304]
Treatment Arm 2 (Time T-1)	−0.478*	[−0.681; −0.275]	−0.444*	[−0.755; −0.133]	0.788*	[0.255; 1.321]
Mediator (Time T-1)	−0.13*	[−0.158; −0.103]	−0.179*	[−0.215; −0.144]	0.607*	[0.515; 0.7]
Outcome (Time T-1)	0.271*	[0.239; 0.304]	0.271*	[0.245; 0.298]	0.263*	[0.226; 0.299]
Covariates						
*Child’s gender*	0.373*	[0.246; 0.501]	0.581*	[0.378; 0.783]	−0.193	[−0.63; 0.243]
*Child’s age*	0.086*	[0.038; 0.134]	0.154*	[0.081; 0.228]	−0.248*	[−0.427; −0.068]
*Child’s orphanhood status*	−0.046	[−0.134; 0.042]	0.004	[−0.129; 0.137]	0.133	[−0.138; 0.403]

**Indirect Effects on Outcome (Time T)**						

**In Treatment Arm 1**						
Outcome (Time T-1)	−0.11*	[−0.173; −0.047]	−0.112*	[−0.195; −0.028]	0.209*	[0.072; 0.346]
Mediator (Time T-1)	−0.032*	[−0.056; −0.007]	−0.039*	[−0.071; −0.006]	0.146*	[0.031; 0.262]
Outcome (Time T-2), Outcome (Time T-1)	−0.03*	[−0.048; −0.012]	−0.03*	[−0.053; −0.008]	0.055*	[0.016; 0.094]
Mediator (Time T-2), Outcome (Time T-1)	−0.009*	[−0.015; −0.002]	−0.011*	[−0.019; −0.002]	0.038*	[0.009; 0.068]
Outcome (Time T-2), Mediator (Time T-1)	0.003	[0; 0.007]	0.003	[0; 0.006]	−0.006	[−0.014; 0.002]
Mediator (Time T-2), Mediator (Time T-1)	−0.027*	[−0.048; −0.006]	−0.033*	[−0.061; −0.005]	0.122*	[0.026; 0.219]
Outcome (Time T-3), Outcome (Time T-2), Outcome (Time T-1)	−0.008*	[−0.013; −0.003]	−0.008*	[−0.015; −0.002]	0.014*	[0.003; 0.026]
Mediator (Time T-3), Outcome (Time T-2), Outcome (Time T-1)	−0.002*	[−0.004; −0.001]	−0.003*	[−0.005; −0.001]	0.01*	[0.002; 0.018]
Mediator (Time T-2), Outcome (Time T-3), Outcome (Time T-1)	0.001	[0; 0.002]	0.001	[0; 0.002]	−0.002	[−0.003; 0]
Mediator (Time T-3), Mediator (Time T-2), Outcome (Time T-1)	−0.007*	[−0.013; −0.002]	−0.009*	[−0.016; −0.002]	0.032*	[0.007; 0.057]
Mediator (Time T-1), Outcome (Time T-3), Outcome (Time T-2)	0.001	[0; 0.002]	0.001	[0; 0.002]	−0.002	[−0.003; 0]
Mediator (Time T-1), Mediator (Time T-3), Outcome (Time T-2)	0	[0; 0.001]	0	[0; 0.001]	−0.001	[−0.003; 0]
Mediator (Time T-1), Mediator (Time T-2), Outcome (Time T-3)	0.003	[0; 0.006]	0.002	[−0.001; 0.005]	−0.005	[−0.012; 0.001]
Mediator (Time T-3), Mediator (Time T-2), Mediator (Time T-1)	−0.023*	[−0.042; −0.005]	−0.028*	[−0.053; −0.004]	0.102*	[0.02; 0.184]
**In Treatment Arm 2**						
Outcome (Time T-1)	−0.13*	[−0.186; −0.073]	−0.121*	[−0.204; −0.037]	0.207*	[0.066; 0.348]
Mediator (Time T-1)	−0.034*	[−0.058; −0.009]	−0.042*	[−0.076; −0.008]	0.148*	[0.029; 0.266]
Outcome (Time T-2), Outcome (Time T-1)	−0.035*	[−0.052; −0.018]	−0.033*	[−0.055; −0.01]	0.054*	[0.015; 0.094]
Mediator (Time T-2), Outcome (Time T-1)	−0.009*	[−0.016; −0.003]	−0.011*	[−0.02; −0.002]	0.039*	[0.009; 0.069]
Outcome (Time T-2), Mediator (Time T-1)	0.004*	[0.001; 0.007]	0.003	[0; 0.006]	−0.006	[−0.014; 0.002]
Mediator (Time T-2), Mediator (Time T-1)	−0.029*	[−0.050; −0.008]	−0.036*	[−0.064; −0.007]	0.123*	[0.024; 0.223]
Outcome (Time T-3), Outcome (Time T-2), Outcome (Time T-1)	−0.01*	[−0.015; −0.004]	−0.009*	[−0.015; −0.002]	0.014*	[0.003; 0.026]
Mediator (Time T-3), Outcome (Time T-2), Outcome (Time T-1)	−0.002*	[−0.004; −0.001]	−0.003*	[−0.006; −0.001]	0.01*	[0.002; 0.018]
Mediator (Time T-2), Outcome (Time T-3), Outcome (Time T-1)	0.001	[0; 0.002]	0.001	[0; 0.002]	−0.002	[−0.003; 0]
Mediator (Time T-3), Mediator (Time T-2), Outcome (Time T-1)	−0.008*	[−0.013; −0.002]	−0.01*	[−0.017; −0.002]	0.032*	[0.007; 0.057]
Mediator (Time T-1), Outcome (Time T-3), Outcome (Time T-2)	0.001	[0; 0.002]	0.001	[0; 0.002]	−0.002	[−0.003; 0]
Mediator (Time T-1), Mediator (Time T-3), Outcome (Time T-2)	0	[0; 0.001]	0	[0; 0.001]	−0.001	[−0.003; 0]
Mediator (Time T-1), Mediator (Time T-2), Outcome (Time T-3)	0.003	[0; 0.006]	0.003	[0; 0.005]	−0.005	[−0.011; 0.001]
Mediator (Time T-3), Mediator (Time T-2), Mediator (Time T-1)	−0.025*	[−0.043; −0.006]	−0.031*	[−0.055; −0.006]	0.103*	[0.02; 0.187]

Notes: All analyses were ran using Mplus 8.9. Regression coefficients were estimated via maximum likelihood with robust estimators (MLR). 95% confidence intervals were estimated via the bias-corrected bootstrap based on 5000 replicate samples.

T1 = baseline; T2 = 12-month follow up; T3 = 24-month follow-up; T4 = 36-month follow-up; T5 = 48-month follow-up.

Confidence intervals which do not include zero are significant at p < 0.05.

## Data Availability

Data will be made available on request.
